# Non-coding RNAs to regulate cardiomyocyte proliferation: A new trend in therapeutic cardiac regeneration

**DOI:** 10.3389/fcvm.2022.944393

**Published:** 2022-08-18

**Authors:** Kele Qin, Xiaohui Xie, Weijie Tang, Danni Yang, Jun Peng, Jianjun Guo, Jinfu Yang, Chengming Fan

**Affiliations:** ^1^Department of Cardiovascular Surgery, The Second Xiangya Hospital, Central South University, Changsha, China; ^2^Hunan Agricultural University, Changsha, China; ^3^Department of Pharmacology, Xiangya School of Pharmaceutical Sciences, Hunan Provincial Key Laboratory of Cardiovascular Research, Central South University, Changsha, China; ^4^Hunan Fangsheng Pharmaceutical Co., Ltd., Changsha, China

**Keywords:** cardiomyocyte, regeneration, ncRNA, miRNA, lncRNA, circRNA

## Abstract

Cardiovascular diseases remain the leading cause of death worldwide, particularly ischemic heart disease (IHD). It is also classified as incurable given the irreversible damage it causes to cardiomyocytes. Thus, myocardial tissue rejuvenation following ischemia is one of the global primary research concerns for scientists. Interestingly, the mammalian heart thrives after an injury during the embryonic or neonatal period; however, this ability disappears with increasing age. Previous studies have found that specific non-coding (nc) RNAs play a pivotal role in this process. Hence, the review herein summarizes the research on cardiomyocyte regenerative medicine in recent years and sets forth the biological functions and mechanisms of the micro (mi)RNA, long non-coding (lnc)RNA, and circular (circ)RNA in the posttranscriptional regulation of cardiomyocytes. In addition, this review summarizes the roles of ncRNAs in specific species while enumerating potential therapeutic strategies for myocardial infarction.

## Introduction

Ischemic heart disease (IHD) is a significant health concern worldwide (1) and has been associated with increased morbidity and mortality over time (2, 3). IHD has also been incurable, given the irreversible damage to cardiomyocytes. In addition, minimal progress for myocardial infarction (MI) treatment on coronary reopening, increased blood flow to the infarcted area, and coronary artery bypass grafts (CABG) usage has been achieved (4–6).

Given that that cardiomyocytes hardly regenerate in mammals distinguishes them from other lower vertebrates (7, 8). However, recent studies have found that cardiomyocytes are not completely non-renewable (4, 9, 10). In contrast, neonatal mouse cardiomyocytes could proliferate to some extent (11, 12). During myocardium development, cardiomyocytes gradually break away from the cell cycle and become non-renewable over time (13, 14).

Cardiomyocytes undergo extensive proliferation during embryogenesis but exit the proliferating cell cycle shortly after birth (9, 13). Most cardiomyocytes undergo the last round of DNA replication and nuclear division without cytokinesis from the 5th day after birth in rodents resulting in approximately 90% of cardiomyocytes stopping proliferation in the 2nd week after birth (15, 16). At this stage (2 weeks after birth), the proliferation potential is limited to a small fraction of mono-nuclear cardiomyocytes (15).

Nonetheless, recent studies have shown that adult cardiomyocytes can re-enter the cell cycle and proliferate under certain mitogen actions (such as fibroblast growth factor, periosteal protein, and neuregulin) (17–20), which seems to be limited to a small number of mono-nuclear cardiomyocytes.

## Characterization of cardiomyocyte proliferation

In the first 10-years of life, the human cardiomyocytes turnover rate, mainly ventricular myocytes, is estimated to be 0.3–1% yearly (21), which is lower than other human body tissues, particularly when tissues are damaged after Mi. Despite several study results indicating that mouse and neonatal fetal hearts myocardium could regenerate after tissue damage (15, 16, 22), G1/S and G2/M cyclin expression and cyclin-dependent kinases in the cell cycle are usually downregulated, while the associated cell cycle inhibitor levels are increased (10, 15).

However, when transitioning between the juvenile or adult phase, cardiomyocytes break out of the cell cycle and enter a quiescence phase (G0) (23). During the MI process or myocardial ischemia-reperfusion (I/R) injury, both regulated and non-regulated cell death processes are involved. Later, scar tissue formation will follow this initial injury response (24). Hence, understanding the heart's regenerative capacity is the primary goal of cardiac regenerative medicine.

### MicroRNA

MicroRNA (miRNA) is an endogenous non-coding single-stranded RNA composed of approximately 20–24 nucleotides (25). It was first described in nematodes in 1993 (25, 26). Since then, miRNA has been investigated in multiple contexts, confirming that it plays a regulatory role in various organisms and associated diseases. The miRNA mechanism binds to the 3' UTR at the end of the messenger RNA (mRNA) and then targets the gene through posttranscriptional regulation, thereby inhibiting the target gene expression in most cases (26). miRNA is widely present in eukaryotes, and there are traces of its existence from lower organisms to humans (8). Its biological characteristics include a high degree of conservation and temporal and tissue expression specificity (27).

With miRNA, there is a high degree of evolutionary conservation among various species with its sequence structure (28). Researchers believe that miRNAs conservation has an important biological significance, suggesting that miRNAs have the same regulatory mechanism during several organisms' development (29). It thus provides additional evidence for the conclusions derived using miRNAs in cross-species studies (28). Likewise, it also provides a basis for the homology of early biological evolution.

**Figure 1:**
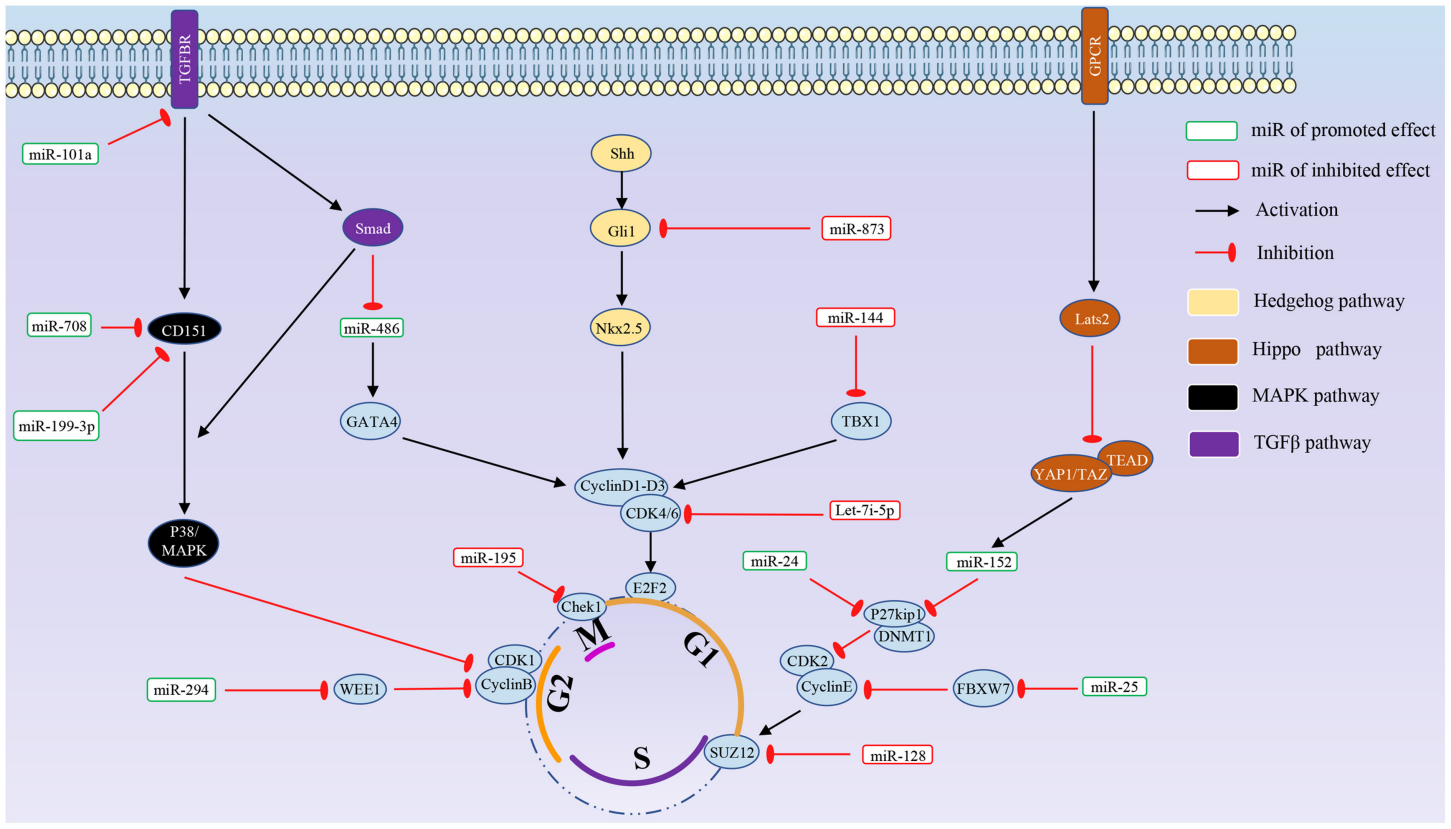
The effects of different miRNAs that directly act on the cell cycle.

**Figure 2:**
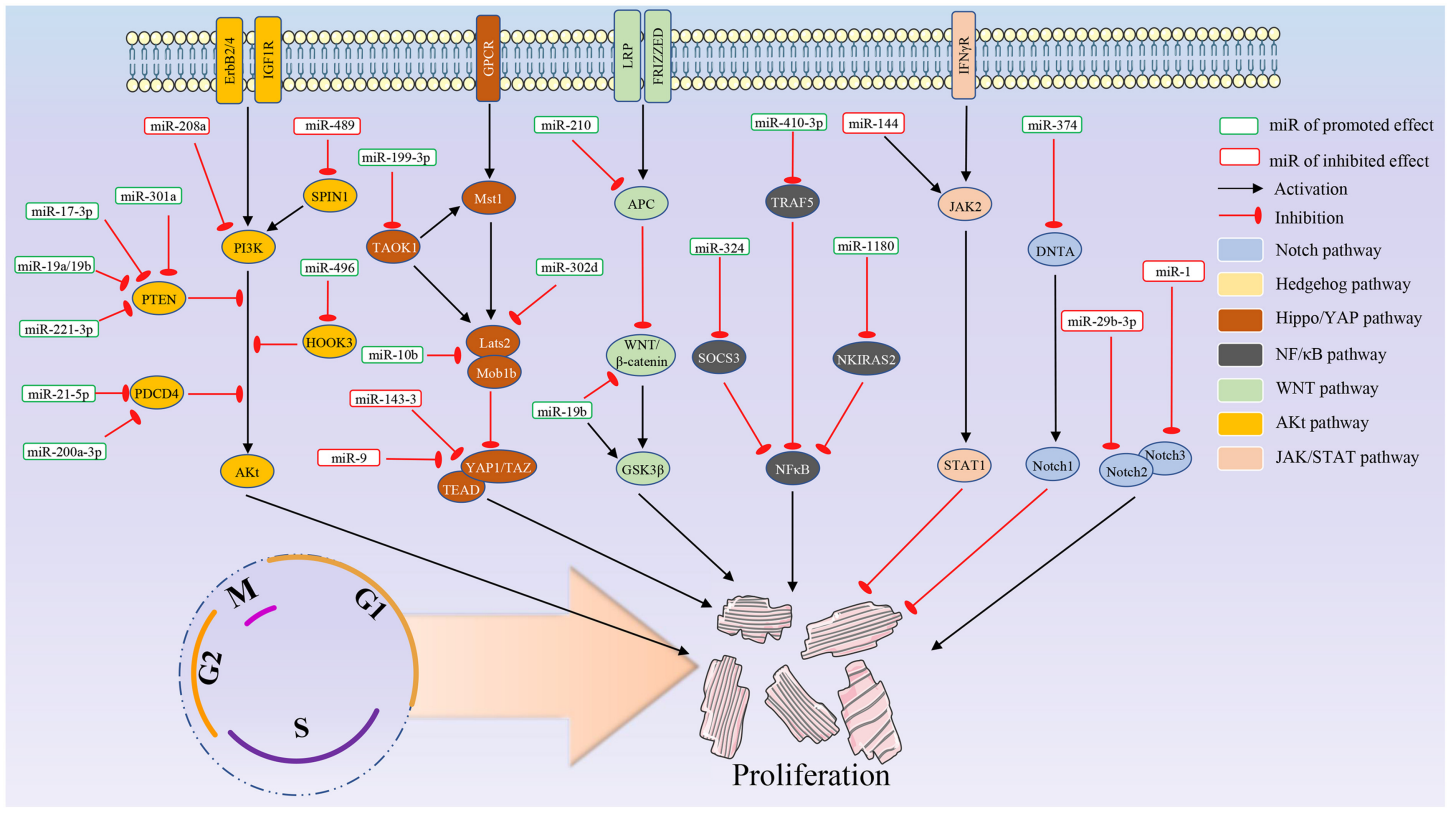
The potential mechanism of the miRNAs on cardiomyocyte proliferation.

In addition, there are significant differences in expression levels for the same miRNA in different tissues and developmental stages (30). Some miRNAs demonstrate temporal expression during development (31). Several studies have pointed out that miRNAs can specifically regulate cell cycle-related proteins, such as CDK and P21, and the proliferation of juvenile cardiomyocytes is also inseparable from the regulation of cyclins and related pathways. miRNAs that directly act on the cell cycle and the relative signal pathway are summarized in Figures 1, 2, respectively.

This review article focuses on the most recent research concerning miRNAs and their role in cardiomyocyte regeneration. miRNAs that enhance or inhibit cardiomyocytes proliferation are summarized in Tables 1, 2.

**Table 1:** A summary of the *in vitro* and *in vivo* effects of miRNAs on enhanced cardiomyocyte proliferation and the potential mechanism.

**miRNA**	**Species *in vitro***	**Species *in vivo***	**Targets**	**Pathway**	**Other effects**	**Reference**
miR-101a	AC16	/	TGFBR1 -	MAPK	Apoptosis -	(32)
miR-10b	hESC-CM	/	LATS1 -	Hippo	Apoptosis -	(33)
miR-1180	Neonatal SD rat ventricular cardiomyocytes	SD rat	NKIRAS2 -	NFκB	Apoptosis -	(34)
miR-133a	AC16	/	/	/	Apoptosis -	(35)
miR-152	Neonatal mice cardiomyocytes	C57BL/6	YAP1- P27kip1 -DNMT1 -	Hippo/YAP	Apoptosis -	(18)
miR-17-3p	Neonatal rat cardiomyocytes H9C2	C57BL/6	TIMP3 -	AKT	Hypertrophy +	(36, 37)
miR-199a-3p	SD NRCM	/	CD151 – P38 -	MAPK	/	(38)
miR-199a-3p	neonatal rats cardiomyocytes	/	TAOK1 (β-TrCP (Cofilin2 -	Hippo	/	(39)
miR-19a/19b	Adult mice cardiomyocytes	C57BL/6	PTEN - BIM -	AKT	Cell death - Apoptosis -	(40)
miR-19b	P19	/	GSK3β + β-catenin -	WNT	Apoptosis -Differentiation +	(41, 42)
miR-200a-3p	AC16	C57BL/6	PDCD4 -	AKT	Apoptosis -	(43)
miR-210	adult Fisher rat cardiomyocytes	C57BL/6	APC - β-catenin +	WNT	cell survival + angiogenesis +	(44)
miR-21-5p	Human cardiomyocytes (HUVECs)	CD1 mice	PDCD4 -	AKT	Apoptosis -	(45)
miR-221-3p	H9C2 HUVECs	SD rat	PTEN -	AKT	Apoptosis -	(46)
miR-24	SD NRCM	SD rat	CDKN1B(p27) -	/	Hypertrophy +	(47)
miR-25	hiPSC-CMs hESC-CMs	Zebrafish	FBXW7 -	/	/	(48)
miR-294	neonatal rat cardiomyocytes	mice	Wee1/CDK1-CyclinB1 axis -	/	/	(49)
miR-301a	neonatal rat cardiomyocytes H9C2	C57BL/6	PTEN -	AKT	Apoptosis -	(50)
miR-302d	hiPSC-CM hESC-CM	/	LATS2 -	Hippo	/	(51)
miR-324	Human neonatal cardiomyocytes	/	SOCS3 -	NFκB	Apoptosis -	(52)
miR-374	ICR mice cardiomyocytes	ICR mice	DTNA -	Notch	Apoptosis -	(53)
miR-486	Embryonic/neonatal mice cardiomyocytes	CD1 mice	GATA4 + FoxO1 - TGFβ/Smad –	TGFβ	/	(54)
miR-496	H9C2	/	HOOK3 -	AKT	Apoptosis -	(55)
miR-708	neonatal rat cardiomyocytes or H9C2	C57BL/6	MAPK14 -	MAPK	Apoptosis -	(56)

**Table 2:** A summary of the *in vitro* and *in vivo* effects of miRNAs on inhibited cardiomyocyte proliferation and the potential mechanism.

**miRNA**	**Species *in vitro***	**Species *in vivo***	**Targets**	**Pathway**	**Other effects**	**Reference**
let-7i-5p	C57BL/6 NMCM	C57BL/6	E2F2 - CCND2 -	/	/	(57)
miR-1	H9C2	/	NOTCH3	Notch	/	(51)
miR-128	neonatal SD rat cardiomyocytes Adult C57BL/6 CMs	C57BL/6	SUZ12 – Cyclin E and CDK2 -	/	/	(58)
miR-143-3p	Neonatal mice cardiomyocytes	C57BL/6	YAP – Ctnd1 -	Hippo	Apoptosis +	(59)
miR-144	H9C2	/	TBX1 -	JAK/STAT	Apoptosis +	(60)
miR-195	C57 Neonatal mice	C57BL/6	Chek1 -	/	/	(61)
miR-208a	SD rat cardiomyocytes	/	PI3K -	PI3K/AKT	Autophagy + Apoptosis +	(62)
miR-29b-3p	HL-1	Zebrafish	NOTCH2	Notch	/	(63)
miR-378a-3p	Neonatal rat cardiomyocytes	C57BL/6	ATg12 – LC3 – P62 +	/	Apoptosis +	(64)
miR-489	H9C2	/	SPIN1 -	PI3K/AKT	Apoptosis +	(65)
miR-612	AC16	/	HOXA13 -	/	Apoptosis +	(66)
miR-873	H9C2	/	GLI1 -	Hedgehog	/	(67)
miR-9	H9C2	/	YAP1 -	Hippo	Apoptosis +	(68)

### miRNAs that enhance cardiomyocyte proliferation

#### Directed at the cell cycle

Researchers found that miR-294 was overexpressed in mouse embryos and was consistent with embryonic heart development. This miR changed rapidly after the heart tissue transitioned from postnatal to adult heart stages (49). After transfection of neonatal rat ventricular myocytes (NRVM) with the miR-294 mimic, CyclinB1, Cdk2, and CyclinE1 expressions were higher than the control cells, indicating that the cells changed to the G1/S phase. The transfected cells had an altered cell cycle and an advantage in energy metabolism. The extracellular acidification rate (ECAR), glycolysis ability, glycolysis, and glycolysis reserve were significantly increased 24 h after miR-294 treatment than the control group.

In addition, the researchers used a targeting scanner to predict the miR-294 target as WEE1. After, luciferase analysis was used to determine if miR-294 bound to the 3' UTR end of WEE1 to regulate NRVM proliferation (49). WEE1, a cell cycle regulatory protein, inactivates the CDK1-CyclinB1 complex and prevents CDK1 phosphorylation at Tyr1530, thus preventing it from entering the G2/M phase (13). Hence miR-294 inhibits WEE1, indicating that miR-294-mediated NRVM cell cycle reentry may be related to CDK1-CyclinB1 complex release and its downstream signal.

P27 is a cyclin-dependent kinase inhibitor that promotes the cell cycle by inhibiting various cyclins and CDK activities so that cells in the G0/G1 phase cannot enter the S phase of the cell cycle. miR-24 expression was higher in transverse aortic constricted (TAC) rats than in sham-operated rats, and researchers speculated that this might be associated with cardiac hypertrophy (47). The target scan predicted that p27 was a potential target in rats and humans. In a subsequent luciferase reporter assay experiment, 3'-UTR bounds to p27 to inhibit P27 activity, which shows that p27 is a miR-24 target.

Likewise, miR-24 can affect cardiac myocyte hypertrophy (47). miR-24, also known as a junctophilin-2 suppressing miRNA (although it could cause cardiac hypertrophy), treatment with antagomir prevents decreased L-type calcium channel/receptor signaling fidelity or efficiency and whole-cell calcium transients. Also, miR-24 decrease shows that suppression of miR-24 could effectively prevent the myocardial transition from compensated to decompensated state (36).

In addition to being a target for miR-24, the P27 is also a target for miR-152. Wang et al. demonstrated that Toll-like receptor 3 (TLR3) promotes myocardial regeneration and repair in neonatal mice, while TLR3-deficient mice showed greater heart function loss and huge infarct size (18). TLR3 ligands can activate the YAP1 pathway in neonatal mouse cardiomyocytes, which can induce cardiomyocyte proliferation. Further experiments confirmed that YAP1 activates miR-152 downstream expression. miR-152 can inhibit p27 and DNMT1 expression and regulate the cell cycle of cardiac myocytes from G1/G0 phase to the S phase (18).

Moreover, miR-25 can promote cardiomyocyte proliferation by targeting the cyclin family of proteins (48). Previous studies showed that cardiomyocytes' ability to proliferate decreased over time in cultured hPSC-CM. In response, investigators examined gene expression at the genome-wide level and screened for significant changes on the sixth and 18th days. Finally, it was determined that miR-25 could significantly promote hESC-CMs and hiPSC-CMs proliferation.

Similarly, potential targets were predicted using a target scan, and finally, miR-25 was reported to target FBXW7 via a dual-luciferase reporter (48). Fbxw7 is a cell cycle regulator that mediates the ubiquitin-dependent proteolysis of several positive cell cycle regulators (69). In addition, miR-25 regulates the FBXW7/CyclinE pathway in cardiomyocyte proliferation, which was observed by RNA sequencing and KEGG enrichment analysis. Finally, *in vivo* experiments in zebrafish confirmed that miR-25 could also regulate FBXW7 to promote proliferation (48) (Table 1).

#### AKT pathway

In 2017, the researchers measured the expression of miR-17-92 cluster members and miRNAs in two different mouse models and concluded that miR-17-3p increased in mouse ischemic/reperfusion injury (IRI) and TAC exercise models (37). The effects on cardiac myocyte hypertrophy, proliferation, and survival were demonstrated *in vitro* and *in vivo*. A target scan was also used to predict the target. After that, the luciferase reporter analysis indicated that metalloproteinase 3 (TIMP3) tissue inhibitor is a direct target gene for miR-17-3p, and phosphatase and angiotensin homolog (PTEN) could be indirectly suppressed by miR-17-3p (37).

These results suggest that miRNA can activate the Akt pathway to achieve cardiac myocyte proliferation. Liu et al. (70) also demonstrated that human serum-derived small exosomes could promote cell proliferation and increase miR-17-3p expression in H9C2 cells, confirming TIMP3 as a direct target gene. Moreover, two other members of the miR-17-92 cluster (miR-19a and miR-19b, which had the same seed sequence) reduced MI size in the MI mouse model, and fractional shortening (FS) was improved. Likewise, adding miR-19a/19b to NRVMs stimulated the proliferation of rat cardiomyocytes *in vitro* (40). Increased expression levels were also detected in patients' blood with dilated cardiomyopathy and coronary heart disease. It thus showed that miR-19a/19b significantly stimulated the proliferation of cardiomyocytes in infancy and adulthood.

In addition, BIM1 and PTEN are both direct targets of miR-19a/19b. miR-19a/19b can also inhibit apoptosis by directly inhibiting apoptosis-related genes (40). miR-301a and miR-221-3p are also direct targets for PTEN (46, 50). Increasing miR-301a expression in H9C2 cells can promote cell proliferation and increase the G1/S transition of the cell cycle. It can also increase the proliferation rate of primary newborn cardiac myocytes. Furthermore, the apoptosis rate of H9C2 cells transfected with miR-301a decreased under hypoxic conditions. Also, miR-301a can increase the proliferation of cardiomyocytes *in vivo*. Further experiments, such as the luciferase reporter assay, showed that the miR-301a could bind to the 3' UTR end of PTEN (50).

Similarly, miR-221-3p from small hMSC exosomes can enhance AKT kinase activity by inhibiting PTEN, thus enabling H9C2 cells to proliferate (46). Researchers have found that the small exosomes released by Young-Exo hMSCs can promote myocardial endothelial cell formation after MI, reduce myocardial fibrosis, inhibit cardiomyocyte apoptosis, and effectively improve cardiac structure and function *in vivo* than Age-Exo cells. Also, after sequencing, it was found that miR-221-3p was upregulated, while miRNA activated the Akt pathway by targeting PTEN (46).

A study by Cheng et al. found that MSC-derived small exosomes were significantly reduced in a rat MI model after treatment, which prevented fibrosis after infarction (71). Using microarray analysis, miR-210 was found to be abundant in MSC-derived small exosomes. With prediction and dual-luciferase analysis, AIFM3 was the downstream target of miR-210. However, the AIFM3 level was up-regulated in hypoxic cardiomyocytes, while p-AKT, PPI3K and p-p53 expressions were down-regulated using western blotting and qPCR analysis. These results suggest that miR-210 may activate the PI3K/Akt pathway (71).

Per bioinformatics analysis and target gene prediction database, Hook3 is a key target gene for Rno-miR-496 and is closely related to cell proliferation. After H9C2 cells transfection with a miR-496 mimic, it was suggested that miR-496 upregulation might be related to H9C2 hypoxia/reoxygenation (H/R-induced apoptosis) and human cardiomyocytes reduction (55). Also, the dual-luciferase reporter assay system confirmed that miR-496 targets Hook3. Subsequently, Hook3 overexpression stimulated apoptosis in H/R-treated cells, thereby inhibiting cell proliferation. miR-496-activated PI3K/Akt signaling was upregulated, while Hook3 showed the opposite trend. Thus, it shows that miR-496 directly regulates apoptosis and the proliferation-related gene expression levels by targeting Hook3 and activating the PI3K/Akt signaling pathway (55).

Programmed cell death 4 (PDCD4) can be involved in various cellular processes, including cell proliferation, autophagy, and apoptosis (43, 72). Researchers have found that it is a direct target gene of several miRNAs and is involved in cardiomyocyte proliferation. Small exosomes isolated from explant-derived cardiac stromal cells from patients with heart failure (FEXO), microRNA array, and PCR analysis showed that miR-21-5p was abnormally expressed in FEXO. miR-21-5p overexpression can restore the impaired cardiac function caused by FEXO (45). Further studies on the mechanism have shown that miR-21-5p inhibited cardiomyocyte apoptosis by targeting PDCD4, promoting angiogenesis by activating the PTEN/Akt signaling pathway, and VEGF expression in vascular endothelial cells.

In addition, Sun and Zhang (43) demonstrated the lncRNA MALAT1 mechanism in myocardial infarction. The study also confirmed that miR-200a-3p could bind to PDCD4, while MALAT1 acts on miR-200a-3p by sponge as the competitive endogenous RNA (ceRNA), upregulating PDCD4 (43). Rescue experiments showed that the MALAT1/miR-200A-3p/PDCD4 axis regulates hypoxia-induced cardiomyocyte proliferation, cell cycle progression, and apoptosis (Figure 1).

#### MAPK pathway

Astragaloside IV (AS/IV), an active component of Astragalus Membranaceous (AM), has been used as one of the traditional drugs to treat cardiovascular diseases. AS/IV can effectively ameliorate myocardial cell injury induced by hypoxia/reoxygenation (H/R), and it has a significant protective effect on myocardial cells after H/R (32). miR-101a expression was increased in H/R cells treated with AS/IV, while dual-luciferase reporter gene analysis showed that miR-101a could target TGFBR1 in H/R cardiomyocytes. Furthermore, AS/IV could promote cell proliferation and upregulate miR-101a expression, thus inhibiting TGFBR1 and TLR2 expression in myocardial cells injured by H/R. Moreover, western blot analysis showed that the downstream genes (p-ERK and p-p38) of the MAPK signaling pathway were inhibited, indicating that AS/IV could inhibit the MAPK signaling pathway of H/R-injured cardiomyocytes (32).

miRNAs, including miR-708, were abundant in embryonic and neonatal cardiomyocytes and less abundant in adult cardiomyocytes (56). Thus, miR-708 overexpression could promote H9C2 cell proliferation or primary cardiomyocytes *in vitro*. miR-708 can also promote myocardial regeneration and cardiac function recovery *in vivo*. In addition, miR-708 protects cardiomyocytes from stress-induced apoptosis under hypoxia or Isoprenaline treatment. Luciferase assay confirmed the direct interaction between miR-708 and MAPK14, which showed that miR-708 partially depended on MAPK14 expression (56). Moreover, miR-199a-3p was a potent activator of rodent cardiomyocyte proliferation. Ying et al. hypothesized that the miR-199a-3p direct target gene was CD151, which activates the negative proliferation regulators by expressing p38, thereby inhibiting the cell cycle (38). Gain-of-function and loss-of-function experiments confirmed that CD151 is essential in inhibiting the cardiomyocyte cell cycle. Thus, it shows that miR-199a-3p achieves cardiomyocyte proliferation by regulating the MAPK pathway.

#### Notch pathway

Zhao et al. discovered the distinct miR-374 expression in a thoracic epidural anesthetic model and a myocardial I/R injured mouse model (53). TEA is a common anesthesia method that can block the sympathetic afferent and efferent nervous systems. Therefore, it is speculated that it may prevent malignant ventricular arrhythmia caused by acute myocardial infarction. In a mouse myocardial I/R injured model, low miR-374 and high DTNA expressions were found. The TEA model has a protective effect on myocardial I/R injury, achieved by increasing miR-374 and decreasing DTNA expression.

The dual-luciferase reporter assay confirmed that DTNA is the target gene for miR-374, and cardiomyocytes overexpressing miR-374 have downregulated DTNA levels that blocked the Notch1 axis. miR-374 overexpression also promoted cardiomyocyte viability, inhibited apoptosis, and increased the cell number arrested in the S phase of the cell cycle. Correspondingly, miR-374 upregulation and DTNA downregulation reduced myocardial infarct size *in vivo*. These results suggest that miR-374 prevents myocardial I/R injury in mice after TEA by inhibiting DTNA-mediated Notch1 axis activity (53).

#### Hippo pathway

Regulating the Hippo signaling pathway to promote endogenous CM proliferation has emerged as a promising strategy for cardiac regeneration (33, 39, 51). LATS1 is the main component of the Hippo pathway. miR-10b was found in human embryonic stem cell-derived CMs (hESC-CMs) and was highly expressed in the early stage, with expression gradually decreasing over time (33). Cell cycle-related genes were higher than the control group, indicating that ESC-CM proliferation was promoted. In addition, ESC-CM transfected with miR-10b also had a significant inhibitory effect on H_2_O_2_-induced apoptosis. Dual-luciferase reporter assay showed that LATS1 was a target for 10b, and the pathway showed that CM proliferation was increased by inhibiting the Hippo pathway (33). Like LATS1, LATS2 is also a component of the Hippo pathway.

Xu et al. studied the fourth day of hPSC differentiation into CM and confirmed that miR-302d expression was significantly reduced (51). miR-302d overexpression significantly promoted hPSC-CMs proliferation. Target scan prediction and luciferase reporter analysis concluded that LATS2 was a direct target gene for miR-302d, promoting CM's proliferation by targeting LATS2 in the Hippo pathway. TAOK1, LATS upstream gene in the Hippo pathway, also has a miRNA targeting effect. miR-199a-3p can target CD151 to activate cardiomyocyte proliferation via the MAPK pathway (38). Thus, it targets the upstream YAP inhibitory kinase (TAOK1) and the E3 ubiquitin ligase (b-TrCP), causing YAP degradation (39), thereby inducing rat cardiomyocytes proliferation.

#### NFκB pathway

Ding et al. investigated the miR-1180 mechanism on cardiomyocytes and found that the miR-1180 observed in the rat's heart at embryonal day 9.5 decreased over time and completely disappeared at the seven postnatal days (34). In H/R-induced cardiomyocytes, miR-1180 expression was downregulated. On the other hand, miR-1180 transfection significantly mimics attenuated myocardial injury and apoptosis, while miR-1180 overexpression promoted cell proliferation through cell cycle processes. In addition, miR-1180 was found to target NKIRAS2, directly regulating the NF-κB pathway. Meanwhile, miR-410-3p attenuated apoptosis in hypoxia-treated cardiomyocytes, while miR-410-3p overexpression promoted hypoxia-preconditioned AC16 cells proliferation (73).

The dual-luciferase assay showed that miR-410-3p could bind to the 3' UTR TRAF5 region. The result was confirmed by inhibiting TRAF5 expression. TRAF5, a member of the tumor receptor-associated factor (TRAF) protein family, is involved in various tumor activities and usually mediates NFκB pathway activation (73). Researchers compared the differentially expressed genes (DEGs) in human myocardial infarction and normal myocardial tissue through the bioinformatics database and identified five hub genes.

One of the genes, SOCS3, was significantly elevated in the MI group (52). Next, it was confirmed by bioinformatics prediction and luciferase reporter assays that miR-324 was its upstream regulatory miRNA, which could both bind to inhibit SOCS3 expression. In *in vitro* experiments, it was clear that miR-324 overexpression promoted cardiomyocyte proliferation, protected cells from apoptosis in the H/R model, and reduced cardiomyocytes, such as TNF-α, p-p65, and p-IκBα. Thus, it showed that miR-324 might improve cardiomyocyte H/R injury by regulating NFκB (52).

#### WNT pathway

miR-19b belongs to the miR-17-92 cluster (41). Previous studies demonstrated that miR-19b could participate in the PTEN/AKT pathway to activate cardiomyocyte proliferation (40), stimulate the WNT pathway, and interact with the gene β-Catenin-related pathway (41, 42). Arif et al. demonstrated (by western blot analysis) that cardiomyocytes in the transfected group had significantly increased proliferation but decreased cell death and downregulated APC (adenomatous polyposis coli) expression. Simultaneously, the group used murine miR-210 to transfect adult rat cardiomyocytes (44). Using a bioinformatics prediction and luciferase reporter assay, Arif et al. confirmed that the APC cell cycle repressor is a direct target for miR-210 in rodents. APC is a WNT signaling pathway antagonist involved in tumor cell migration, adhesion, and apoptosis (74). These showed that miR-210 overexpression rescued cardiac function after cardiac injury in adult mice by promoting CM proliferation, cell survival, and angiogenesis.

#### Others

Physiologically, the ventricular myocytes are stretched when the heart beats normally. Researchers hypothesized that the cardiomyocyte stretch-response pathways are closely related to cardiac development and that perturbations in biomechanical stimuli may cause conditions such as hypoplastic left heart syndrome (HLHS) (54). miR-486 expression was increased in cardiomyocytes under cyclic stretch and in HLHS patients with increased right ventricular (RV) stretch *in vivo*. Furthermore, increasing miR-486 for 3 days in neonatal mice was sufficient to promote ventricular growth and cardiomyocyte proliferation significantly.

Subsequent studies confirmed that miR-486 inhibited TGF-β/Smad signaling to promote cardiomyocyte proliferation. Yang et al. found that with hypoxia prolongation, lncRNA TUG1 and miR-133a exhibited time-dependent variation. Both expressions were complementary, meaning TUG1 expression was increased while miR-133a expression gradually decreased (35). TUG1 silencing enhanced miR-133a and miR-133b expression. A luciferase reporter assay was subsequently performed, indicating that miR-133a is a direct target for TUG 1 in AC16 cells. In addition, TUG1 acts as a competitive endogenous RNA (ceRNA) to inhibit miR-133a expression. Hence, it shows that miR-133a can promote AC16 cell proliferation and inhibit cell apoptosis (Table 1).

### miRNAs that inhibit the cardiomyocyte proliferation

#### Directed at the cell cycle

Let-7i-5p affects cardiomyocyte cell proliferation and interferes with cardiomyocyte repair after injury (57). Let-7i-5p transfection mimics in mouse cardiomyocytes can significantly reduce cardiomyocyte count, indicating that let-7i-5p inhibits proliferation. Additionally, target scan prediction and luciferase reporter assay found that let-7i-5p is a target relationship with E2F2 and CCND2, which binds their 3' UTR end and inhibits E2F2 and CCND2 proteins expression (57). Huang et al. observed that miR-128 is upregulated in murine cardiomyocytes during the postnatal period, switching from proliferation to terminal differentiation (58). Furthermore, miR-128 overexpression impairs CM proliferation and cardiac function by downregulating SUZ12 expression. The outcome could suppress cyclin-dependent kinase inhibitor-p27 expression and activate the positive cell cycle regulators Cyclin E and CDK2.

miR-195 and its expression, a member of the miR-15 family, gradually decreased over time in neonatal mouse ventricular myocytes. Also, the expression level was almost 6-fold higher on the 10th day of life than on the 1st day of life (61). miR-195 was also confirmed to affect cardiomyocyte proliferation *in vitro* and *in vivo*. Subsequently, in the study of target genes, it was found that miR-195 can bind to checkpoint kinase 1 (Chek1) and inhibit the increase in cardiomyocyte count by affecting the cardiomyocyte cell cycle normal operation. Chek1, one of the key regulatory enzymes of the cell cycle, is mainly involved in regulating mitotic stages and coordinates progression via the G2/M, spindle checkpoints, chromosome segregation, and cell division (75). Therefore, miR-195 primarily regulates mouse cardiomyocyte proliferation by affecting Chek1 expression (Table 2).

#### JAK-STAT pathway

Cao et al. studied the miR-144 effect on rat cardiomyocytes and confirmed that it is essential in regulating cardiomyocyte proliferation (60). miR-144 inhibits cell proliferation by blocking H9C2 cells in the G1 phase. Bioinformatics prediction and luciferase reporter gene analysis indicated that miR-144 directly targets TBX1. miR-144 and TBX1 co-overexpression promotes the transition from the G1 to the S phase, upregulates cell proliferation, and downregulates apoptosis by inhibiting the JAK2/STAT1 signaling pathway.

#### Hedgehog pathway

Researchers found that the miR-873 expression was upregulated in patients' serum with congenital heart defects (67). miR-873 overexpression can inhibit H9C2 cell proliferation and induce cell cycle arrest, which clarifies miR-873 role in cardiomyocytes. miR-873 has a predicted target site in the 3'-untranslated region (3'-UTR) of GLI1, as shown by the bioinformatics algorithm and verified by the dual-luciferase reporter experiment (67). Glioblastoma-associated oncogene 1 (Gli1) is a nuclear effector of the Hedgehog pathway and plays an essential role in embryonic development and organogenesis.

#### AKT pathway

Similar to the TEA model, sevoflurane is a common gas anesthetic that blocks the sympathetic (afferent/efferent) effects and may reduce myocardial infarction (53). Shi et al. studied the protection by sevoflurane in a rat cardiomyocyte IRI model. They concluded that sevoflurane application could effectively reduce autophagy and apoptosis-related cytokines expression in IRI cells, while miR-208a could reverse sevoflurane effects on IRI cells (62). The induced effect reduces cardiomyocyte proliferation and arrests the cardiomyocytes in the G0/G1 phase. When studying the pathway, the PI3K and AKT mRNA expressions were significantly increased in the post-treatment group with sevoflurane, while sevoflurane-preconditioning combined with the miR-208a inhibitor increased PI3K and AKT mRNA levels.

It, therefore, suggests that miR-208a low expression can activate the PI3K/AKT signaling pathway and inhibit the expression of the autophagy-related factors in myocardial SI/RI cells (62). miR-489 can be highly expressed in H/R-treated rat cardiomyocytes, and miR-489 overexpression can accelerate its apoptosis. Chen et al. found that the SPIN1 gene is a downstream target gene for miR-489, and this miRNA can be combined with its 3' UTR to downregulate SPIN1 expression. Additionally, western blots showed that miR-489 overexpression inhibited the PI3K/AKT pathway (65). Thus, miR-489 could induce cardiomyocyte apoptosis by inhibiting the AKT pathway (Figure 2).

#### Notch pathway

Yang et al. applied next-generation sequencing (NGS) to screen patients' entire miRNA expression profile with congenital heart disease (63). It was found that the of miR-29b-3p expression in the patients' right ventricular outflow tract (RVOT) was significantly increased, while NOTCH2 was downregulated in the RVOT. *In vivo* experiments using miR-29b-3p mimics injected into zebrafish embryos caused several malformations, including corporeal and cardiac malformations. *In vitro* experiments, where miR-29b-3p was transfected into HL1 cells showed that the expression of the proliferation-related proteins was downregulated, while expression was upregulated in the miR-29b-3p inhibitor group.

On the other hand, luciferase reporter analysis indicated that miR-29b-3p was an upstream regulatory miRNA for NOTCH2, indicating that miR-29b-3p could regulate the NOTCH pathway and inhibit mouse cardiomyocyte proliferation (63). Meanwhile, miR-1 was confirmed to regulate the NOTCH pathway and inhibit cardiomyocyte proliferation (76). In rat cardiomyocytes, hypoxia inhibited cell proliferation but promoted cell apoptosis and miR-1 expression, while miR-1 expression downregulation had the opposite effect. Subsequently, bioinformatics analysis, luciferase reporter, and RNA immunoprecipitation assays indicated that NOTCH3 was a direct target for miR-1. Upregulation was shown to reverse the miR-1 effects on the proliferation, apoptosis, and autophagy of the hypoxia-treated H9C2 cells (76).

#### Hippo pathway

Ma et al. studied the melatonin effect on mice cardiomyocytes and found that it could significantly reduce oxidative stress and apoptosis in cardiomyocytes after myocardial infarction (59). *In vitro*, melatonin stimulated neonatal mouse cardiomyocytes to re-enter the cell cycle and significantly inhibited miR-143-3p levels. In contrast, miR-143-3p overexpression inhibited melatonin-induced cardiomyocyte mitosis. Subsequent experiments proved that miR-143-3p target genes were YAP and Ctnd1, suggesting that miR-143-3p could regulate the YAP signaling pathway under the influence of melatonin and promotes cellular regeneration in myocardial infarction (59). With hypoxia prolongation in H9C2 cells, miR-9 expression was upregulated, while YAP1 expression was downregulated. miR-9 knockdown increased the hypoxic H9C2 cells' proliferation and inhibited apoptosis and caspase 3/7 activity, while miR-9 overexpression had the opposite effect on hypoxic H9C2 cells (68).

In addition, a luciferase reporter showed that YAP1 is a direct target for miR-9. YAP1 gene knockdown inhibited the hypoxia-exposed H9C2 cells proliferation and promoted apoptosis. Also, the YAP1 knockout attenuated the anti-miR-9 effects on the proliferation and apoptosis of the hypoxic H9C2 cells. The results showed that in hypoxia-exposed H9C2 cells, miR-9 increased cardiomyocyte apoptosis and inhibited cardiomyocyte proliferation by affecting the YAP pathway.

#### Others

Li et al. investigated patients with chronic heart failure (CHF) and compared these patients with a normal control group. The result showed that the lncRNA LUCAT1 in blood was reduced by 1.7 times, suggesting that it may be related to prognostic factors (66). LUCAT1 inhibitor transfection into the AC16 cells can accelerate cardiomyocytes' apoptosis. The study also found that miR-612 expression was increased. Thus, follow-up experiments determined that LUCAT1 can compete with miR-612 as a ceRNA in AC16 cells, showing that they are directly related. Simultaneously, the HOXA13 target for miR-612 was also identified. Rescue experiments showed that the lncRNA LUCAT1 promotes cell proliferation and inhibits apoptosis in CHF patients through via miR-612/HOXA13 axis (66).

LncRNA NEAT1 is involved in various disease progression, but the mechanism involved in myocardial infarction is still unclear. LncRNA NEAT1 expression in more than 100 patients' blood with unstable angina pectoris and ischemic cardiomyopathy/MI was significantly higher than the normal controls (64). After the I/R injury model was established *in vivo*, the NEAT1 level significantly increased over time. NEAT1 knockdown ameliorated hypoxia-induced cardiomyocyte injury. NEAT1 was subsequently predicted to be a target for miR-378a-3p, where subsequent luciferase assay results showed that the miR-378a-3p expression significantly reduced the NEAT1 reporter gene (wild-type non-mutated) luciferase activity (64). It was also noted that the NEAT1/miR-378a-3p axis existed in rat cardiomyocytes under hypoxia (Table 2).

### LncRNAs that inhibit cardiomyocyte proliferation

In the mammalian genome sequence, 4%−9% of transcripts sequentially produced are lncRNAs, with the corresponding proportion of protein-coding RNA being approximately 1%. Though lncRNAs were thought to be secondary RNA polymerase-II-transcription products with no biological functions (77), long non-coding RNAs (lncRNAs) are a cluster of functional non-translated RNA molecules with a length >200 nucleotides. Recent studies have shown that lncRNAs are widely involved in several critical regulatory processes, such as chromosome silencing, genome imprinting, chromatin modification, transcription activation, transcription interference, and nuclear transport (78, 79). Major LncRNAs are classified into four types per their position in the genome relative to the protein-coding gene, including Antisense lncRNAs, Bidirectional lncRNAs, Intergenic lncRNAs, and Sense-intronic lncRNAs. (17, 80, 81). LncRNAs play an essential role in regulating cardiomyocyte proliferation by enhancing or suppressing cell cycle progression.

On the one hand, some lncRNAs act as sponges for inhibitory miRNAs to inhibit cardiomyocyte proliferation. The lncRNA AZIN2-sv, AZIN2 gene splicing variant, directly binds to miR-214 and acts on the PTEN target gene. In addition, AZIN2-sv inhibit the AKT pathway by blocking its inhibitory effect on PTEN. Therefore, AZIN2-sv loss can promote cell survival and proliferation (80). Similarly to AZIN2-sv, the lncRNA CRRL binds to miR-199a-3p and thereby increases the target gene expression of Hopx, a critical negative regulatory factor of CM proliferation, suppressing CM proliferation (82). However, the lncRNA CAREL acted as a ceRNA for miR-296 to downregulate Trp53inp1 and Itm2a, the miR-296 target gene. The Trp53inp1 has been found to cause cell cycle arrest in G1 and to increase p53-mediated apoptosis, while Itm2a was associated with G2/M cell cycle arrest as a tumor suppressor. Hence, CAREL negatively controls cardiomyocyte proliferation.

Cardiac-specific CAREL overexpression in mice reduced cardiomyocyte division and proliferation and blunted neonatal heart regeneration after injury. Similarly, CAREL reduced human-induced pluripotent stem cell-derived cardiomyocyte proliferation (83). LncRNA-specific transcript 5 (GAS5) expression affects cardiomyocyte proliferation, cell cycle, and apoptosis. LncRNA Gas5 regulates myocardial infarction by targeting the miR-525-5p/CALM2 axis. Studies have shown that Gas5 and CALM2 expression in cardiomyocytes after infarction is significantly upregulated, while miR-525-5p expression is significantly downregulated. Gas5 and CALM2 overexpression significantly promotes cardiomyocyte apoptosis and inhibits cardiomyocyte proliferation (84).

In addition, Gas5 stimulates PDCD4, inhibiting the PI3K/AKT signal pathway. LncRNA-Gas5 regulates the PDCD4 expression by targeting miR-21, mediating myocardial infarction-induced cardiomyocyte apoptosis. Thus, it implies that Gas5 may become a therapeutic target for MI (72). However, LncRNA GAS5 inhibits NLRP3 (inflammasome) activation-mediated pyroptosis in diabetic cardiomyopathy by targeting miR-34b-3p/AHR. GAS5 acts as a competing endogenous RNA to enhance AHR expression by sponging miR-34b-3p and repressing NLRP3 (inflammasome) activation-mediated pyroptosis to improve diabetic cardiomyopathy (85). Additionally, lncRNA XIST overexpression reduces cardiac regeneration and promotes apoptosis by negatively targeting the miR-130a-3p/PDE4D axis (86).

On the other hand, some lncRNAs directly act on mRNAs and proteins or act as guides for chromatin modifiers to supress cardiomyocyte proliferation. The LncRNA CPR targets minichromosome maintenance 3 (MCM3), a DNA replication promoter, and cell cycle progression to inhibit cardiomyocyte proliferation. CPR- knocked down cardiomyocytes and CPR-knocked out mice exhibit increased CM renewal after MI (87). Moreover, the lncRNA TUC40- overexpression reduced the target gene Pbx1 expression, cardiomyocyte induction and differentiation, inhibited proliferation and promoted apoptosis. Proliferation was possibly inhibited due to G2/M cell cycle arrest and the increased induced apoptosis rate (88).

### LncRNAs that stimulate cardiomyocyte proliferation

Some lncRNAs promote cardiomyocyte proliferation apart from lncRNAs that suppress cardiac regeneration. A novel upregulated fetal lncRNA ECRAR promoted DNA synthesis, mitosis, and cytokinesis in the seventh post-natal day and adult rat cardiomyocytes. ECRAR was transcriptionally upregulated by E2F transcription factor 1 (E2F1). After that, ECRAR bound and promoted extracellular signal-regulated kinases 1 and 2 (ERK1/2) phosphorylation, causing cyclin D1 and cyclin E1 downstream target activation, lastly activating E2F1. The E2F1-ECRAR-ERK1/2 signaling formed a positive feedback loop to drive cell cycle progression and promote CM proliferation (89). Moreover, antisense lncRNA, silent information regulator enzyme 1 (Sirt1), has been shown to enhance cardiomyocyte proliferation and synchronously attenuate cardiomyocyte apoptosis (80). Unlike other lncRNAs, Sirt1 antisense lncRNA can bind the Sirt1 3'-UTR, enhancing Sirt1 stability and increasing Sirt1 abundance at both the mRNA and protein levels.

Sirt1 was involved in Sirt1 antisense lncRNA-induced cardiomyocyte proliferation (80). The LncRNA NR_045363 was primarily expressed in cardiomyocytes and rarely in non-cardiomyocytes. NR_045363 overexpression in a 7-day-old mice heart could improve cardiac function and stimulate cardiomyocyte proliferation after myocardial infarction (90). lncRNAs that regulate cardiomyocyte proliferation are summarized in Table 3.

**Table 3:** A summary of the lncRNAs on cardiomyocyte proliferation and the potential mechanism.

**LncRNA**	**Effect on proliferation**	**Sponged by**	**Target gene**	**Pathway**	**Reference**
AZIN2-sv	Inhibit	miR-214	PTEN	AKT	(66)
CRRL	Inhibit	miR-199a-3p	Hopx	/	(82)
CAREL	Inhibit	miR-296	Trp53inp1/ Itm2a	/	(83)
GAS5	Inhibit	miR-525-5p	CALM2	/	(84)
GAS5	Inhibit	miR-21	PDCD4	PI3K/AKT	(72)
GAS5	Inhibit	miR-34b-3p	AHR	/	(76)
XIST	Inhibit	miR-130a-3p	PDE4D	/	(86)
CPR	Inhibit	/	MCM3	/	(87)
TUC40-	Inhibit	/	Pbx1	/	(91)
ECRAR	Promote	/	ERK1/2	/	(65)
Sirt1	Promote	/	Sirt1 3'-UTR	/	(91)
NR_045363	Promote	/	-	/	(90)

### CircRNA and cardiomyocytes

CircRNA is a single-stranded RNA type connected end-to-end for specific functions. It implies that the 3' and 5' ends of the RNA form a covalent bond. CircRNA is less prone to degradation by exonucleases than conventional linear RNA because it lacks of 5' and 3' tails (92). In the early 1990's, an accidental PCR amplification revealed circRNA to the world (93). 2 years later, scientists discovered the biological behavior of circular transcription while studying the key mouse gene Sry, which is dominant in sex determination during embryonic development (94).

Following extensive development and popularization of RNA sequencing technology, several achievements have been made in circRNA. Several experiments have confirmed that certain circRNAs have been proven to encode proteins, given that numerous circRNA types come from protein-coding genes (95, 96). However, the biological functions of most circular RNAs are still unclear. This article mainly introduces the relationship between circRNA, cardiomyocyte proliferation, and circRNA biological effects.

CircRNAs (circular RNAs) are emerging as powerful cardiac development regulators and diseases that regulate cardiac regeneration. CircRNA Nfix (circNfix) was overexpressed in humans, rats, and mice in the adult heart. Experiments *in vitro* and *in vivo* indicated that cardiomyocyte proliferation was increased by circNfix knockdown, whereas it was inhibited by circNfix overexpression. Mechanistically, super-enhancer-regulated circNfix inhibits cardiac regenerative repair and functional recovery after myocardial infarction by suppressing Ybx1 ubiquitin-dependent degradation and increasing miR-214 activity after MI (97).

CircHIPK3 inhibits proliferative ability and stimulates human-derived cardiomyocyte apoptosis after myocardial Ischemia-reperfusion injury by binding to miRNA-124-3p, which aggravates myocardial ischemia-reperfusion injury (98). However, another study demonstrated that circHIPK3 was overexpressed in fetal and neonatal mice hearts. CircHIPK3 promotes division and mouse cardiomyocyte proliferation by increasing the Notch1 intracellular domain (N1ICD) acetylation, significantly increasing its stability and reducing degradability. In addition, circHIPK3 acted as a sponge for miR-133a to promote connective tissue growth factor (CTGF) expression, activating endothelial cells and improving cellular function (99).

Notwithstanding, various circRNAs also show the ability to regulate the proliferation of cardiomyocytes during myocardial I/R injury positively. CircRNA-68566 participated in I/R by regulating the miR-6322/PARP2 signaling pathway, which binds to miR-6322 to enhance proliferation (100). circSNRK overexpression could also facilitate the proliferation of cardiomyocytes. In the post-infarction area induced by adeno-associated virus 9 (AAV9), circSNRK could phosphorylate GSK3β activity and miR-103-3p target, while circSNRK can act as a sponge for miR-103-3p to promote cardiomyocytes regeneration (101).

However, other researchers suggested overexpressed CircANXA2 could inhibit the H/R-treated H9C2 cell proliferation. Also, further investigation showed that CircANXA2 could be a sponge for miR-133, reversing the inhibition of proliferation and increasing apoptosis (102). Moreover, circRNA PVT1 (circPVT1) can act as a sponge for two miRNAs (miR-125b and miR-200a), increasing cell apoptosis and blocking cardiomyocyte proliferation *in vitro*. Luo et al., demonstrated that silencing the circPVT1 expression could prevent heart I/R injury in rats and restore cardiomyocyte viability by regulating the circPVT1/miR-125b/miR-200a axis (103). circRNAs that regulate cardiomyocyte proliferation are summarized in Table 4.

**Table 4:** A summary of the circRNAs on cardiomyocyte proliferation and the potential mechanism.

**CircRNA**	**Effect on proliferation**	**Sponged by**	**Target gene**	**Other effects**	**Reference**
CircNfix	Promote	miR-214	Ybx1 ubiquitin-dependent degradation	/	(58)
CircHIPK3	Inhibit	miR-124-3p	/	Induces apoptosis	(98)
	Promote	miR-133a	N1ICD acetylation CTGF	Coronary vessel endothelial cell proliferation (migration (and tube-forming capacity and subsequent angiogenesis.	(99)
Circ68566	Promote	miR-6322	PARP2	/	(72)
CircSNRK	Promote	miR-103-3p	GSK3β Phosphorylation	Reduced cardiomyocyte apoptosis enhanced angiogenesis	(101)
CircANXA2	Inhibit	miR-133	/	Induces apoptosis	(102)
CircPVT1	Inhibit	miR-125b 200a	P53 SIRT7 Keap1 PDCD4	/	(103)

### ncRNAs as biomarkers and therapeutic targets

Cardiovascular diseases (CVD) are the main cause of morbidity and mortality worldwide. The adult heart cannot regenerate new cardiomyocytes during myocardial infarction and heart failure. In recent years, increasing evidence suggests that circulating ncRNAs can serve as diagnostic biomarkers and potential new therapeutic targets for several cardiovascular disease, especially myocardial infarction.

MicroRNAs are considered ideal therapeutic targets in myocardial infarction. In a reperfused AMI porcine model, a single microencapsulated anti-miR-92a intracoronary injection restrained left ventricle remodeling without adverse effects (104). Likewise, the systemic injection of a locked nucleic acid-modified anti-miR-15 effectively rendered cardiomyocytes resistant to hypoxia-induced cardiomyocyte death (105). Additionally, a miR-146a mimic selective administration reproduces some benefits for cardiosphere-derived cells (CDC) exosomes.

The findings identified exosomes as key mediators for CDC-induced regeneration and highlighted exosomes' potential utility as cell-free therapeutic candidates (104). Another study indicated that exosome cargo analysis in mice and humans identified conserved pro-regenerative miRs, which recapitulated the therapeutic effects of promoting cardiomyocyte proliferation (106). In summary, these studies show that miRNA-based therapy using modified oligonucleotides is a promising therapeutic agent for patients suffering from massive acute myocardial infarction or affecting cardiac remodeling and protecting cardiac function after ischemic injury.

Recent advances involving targeting miRNAs, while lncRNAs, a novel and challenging class of potential drug targets will be given priority. A study that collected plasma from patients with cardiac remodeling after acute myocardial infarction and performed transcriptome analysis identified LIPCAR as a potential biomarker for heart failure. In addition, LncRNA LIPCAR (long intergenic non-coding RNA predicting cardiac remodeling) is associated with cardiovascular death in patients with heart failure and a prognostic indicator in patients with heart failure (107). Another study found that LncRNA ANRIL and KCNQ1OT1 levels could predict the degree of left ventricular dysfunction (108). Recent studies have shown that LncRNAs CoroMarker and LncPPARδ are predictive markers for coronary heart disease (109). Despite these advances, circulating ncRNAs cellular origin is largely unknown, and little is currently known about the causality of the underlying disease.

## Conclusions

Microscopically, heart failure caused by the decrease in absolute cardiomyocyte count is still the most crucial factor threatening human life. In addition, current treatment methods cannot supplement/replenish cardiomyocytes, the root cause of failed treatments. The present review collected and summarized information showing that cardiomyocytes, particularly during the neonatal or fetal period, have an inherent ability to proliferate, although gradually inhibited by different degrees of extracellular or intracellular factors.

The nature and importance of these stimuli and how they affect the myocardial cell cycle are still the subject of in-depth research. Starting with lower vertebrates and rodents is essential and effective in understanding the human heart regenerative ability in the evolutionary process and the corresponding cellular mechanism regulation. Notably, the cardiomyogenesis regulatory mechanisms identified in these animal models appear to function similarly in humans. Cell sequencing technologies will make large-scale target identification possible, and new gene therapy technologies will provide new tools for precise regulation *in vivo*.

From the current research, various ncRNAs, including miRNA, lncRNA, and circRNA, can control the essential genes and regulatory pathways of various cellular processes. Most non-coding RNAs in current research directly or indirectly act in a regulatory role on cell cycle-associated proteins. Therefore, enabling cardiomyocyte's' reentry into the cell cycle would be a crucial breakthrough. The studies on miRNAs in this research field of cardiomyocyte regeneration are emerging compared with other ncRNAs. Although lncRNA and circRNA are novel and unexplored, the mechanisms behind them seem to be largely inextricably linked to miRNA regulation. Hence fully understanding the miRNAs mechanism may still allow breakthroughs in solving myocardial regeneration issues. Although there is progress non-coding RNA usage for cardiomyocyte proliferation and repair, there are still significant challenges in the actual combination of clinical guidance and treatment.

## Author contributions

KQ drafted the manuscript and were responsible for the collection of data or analysis. CF designed the study. WT, DY, JY, JP, and JG revised the manuscript. All authors read and approved the final manuscript.

## Funding

This work was supported by the Science and Technology Innovation Program of Hunan Province (2021RC2106 to CF) and the Natural Science Foundation of Hunan Province (2022JJ20088 to CF).

## Conflict of interest

Authors JG and CF were employed by the company Hunan Fangsheng Pharmaceutical Co. Ltd. The remaining authors declare that the research was conducted in the absence of any commercial or financial relationships that could be construed as a potential conflict of interest.

## Publisher's note

All claims expressed in this article are solely those of the authors and do not necessarily represent those of their affiliated organizations, or those of the publisher, the editors and the reviewers. Any product that may be evaluated in this article, or claim that may be made by its manufacturer, is not guaranteed or endorsed by the publisher.

## Abbreviations

circRNA: circular RNA
CeRNA: competitive endogenous RNA
ECAR: Extracellular Acidification Rate
ESC: Embryonic Stem Cell
HLHS: Hypoplastic Left Heart Syndrome
IHD: Ischemic Heart Disease
iPSC: induced Pluripotent Stem Cell
I/R injury: Ischemic-Reperfusion injury
lncRNA: long non-coding RNA
miRNA: micro RNA
MI: Myocardial Infarction
mRNA: messenger RNA
MSC: Mesenchymal Stem Cell
ncRNA: non-coding RNA
NRVMs: Neonatal Rat Ventricular Myocytes
TAC: Transverse Aortic Constriction
TEA: Thoracic Epidural Anesthesia
UTR: Untranslated Region.
